# Gonadal mosaicism mediated female-biased gender control in mice

**DOI:** 10.1007/s13238-022-00910-w

**Published:** 2022-03-25

**Authors:** Meizhu Bai, Dan Liang, Yan Cheng, Guolong Liu, Qiudao Wang, Jinsong Li, Yuxuan Wu

**Affiliations:** 1 State Key Laboratory of Cell Biology, Shanghai Key Laboratory of Molecular Andrology, CAS Center for Excellence in Molecular Cell Science, Shanghai Institute of Biochemistry and Cell Biology, Chinese Academy of Sciences, University of Chinese Academy of Sciences, Shanghai, 200031 China; 2 Shanghai Frontiers Science Center of Genome Editing and Cell Therapy, Shanghai Key Laboratory of Regulatory Biology, Institute of Biomedical Sciences and School of Life Sciences, East China Normal University, Shanghai, 200241 China; 3 Department of Obstetrics and Gynecology, The First Affiliated Hospital of Anhui Medical University, Hefei, 230022 China; 4 NHC Key Laboratory of Study on Abnormal Gametes and Reproductive Tract (Anhui Medical University), Hefei, 230032 China; 5 Key Laboratory of Population Health Across Life Cycle (Anhui Medical University), Ministry of Education of the People’s Republic of China, Hefei, 230032 China

**Dear Editor,**

In nature or during artificial breeding, evolutionary intervention or desirable trait selection could be achieved by means of gender control in an organism. Previously reported approaches including sex-sorted semen and genetic manipulation of a certain gene (Holden and Butler, 2018; Yosef et al., 2019). In mice, a recent study demonstrated a genetic system for biasing sex ratio through crossing two genetically engineered mouse lines (Yosef et al., 2019). The maternal line encodes functional Cas9 protein on an autosomal chromosome, whereas the paternal encodes guide RNAs on Y chromosome targeting vital genes. After fertilization, males carrying both Cas9 and guide RNAs were self-destructed, resulting in a female biased sex ratio. However, the litter size is proximate half of normal size since half of the progeny are eliminated. Moreover, a reproductive reservoir of males and females must be maintained for crossing. Here, we established a mouse line carrying *Hspa2* promoted Cas9 and constitutively expressed sgRNA targeting repetitive sequences on Y chromosome. In those mice, spermatogenic cells carrying Y chromosome were eliminated during spermatogenesis, resulting in female biased sex ratio in progenies whereas the total number was unaffected.

The male-specific region of mouse Y chromosome (MSY) are composed of multiple DNA repeats (Cocquet et al., 2009). *Sly*, *Ssty1*, *Ssty2* as multicopy genes with highly repetitive DNA sequences, are located at the long arm of mouse Y chromosome (MSYq) (Fig. 1A) (Toure et al., 2004). To target the repetitive sequence on Y chromosome via CRISPR/Cas9, firstly we designed three sgRNAs targeting each gene, with each sgRNA recognizing more than 100 copies of DNA on MSY (Fig. 1B). Meanwhile, male or female mouse embryonic stem cell (ESC) lines carrying constitutively expressed Cas9 were generated through blastocysts derivation, followed by Cas9-expressing lentivirus infection (Fig. S1A and S1B). Next, all the lentiviral sgRNAs targeting repetitive sequence on Y chromosome were packaged and infected into each male or female mESC line, respectively. Around 72 h after infection, cell viability was analyzed through cell counting. The result indicated that male mESCs, compared with female showed significantly decreased cell survival (Fig. 1C; Table S1). In addition, cell viability varied among different Y chromosome targeting sgRNAs, which may be associated with respective mutation efficiency. To prove that the variation of cell viability between male and female mESC lines were Y chromosome associated, we additionally designed 3 sgRNAs targeting repetitive sequences on autosome or X chromosome and 2 sgRNAs targeting a unique locus on *Rosa26*. For each sgRNA targeting autosome or X chromosome, there are more than 100 copies of target and none of them are essential genes or chromosome structure sequences related (Fig. 1B). When transduced these additional sgRNAs into male or female mESCs through lentivirus infection, we found that at 72 h post infection, both male and female mESCs carrying sgRNA targeting autosome or X chromosome died, while mESCs carrying sgRNA targeting *Rosa26* proliferate normally, regardless of gender (Fig. 1C; Table S1).

**Figure 1:**
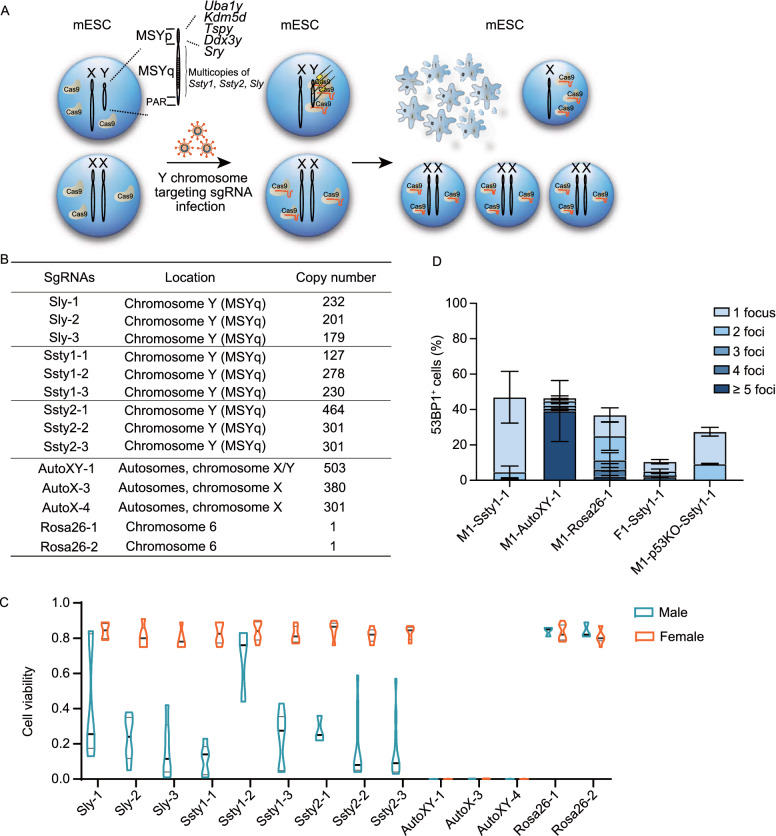
**Multiple DNA DSBs induced by CRISPR/Cas9 trigger cell death in mESCs**. (A) Schematic of male mESC death induced by CRISPR/Cas9 mediated multiple DNA DSBs on Y chromosome. (B) SgRNAs targeting multi-loci on Y chromosome, autosome or X chromosome and sgRNAs targeting single locus on *Rosa26* gene. (C) Statistics of cell viability at 72 h post infection of lentiviral sgRNA. (D) Quantification of 53BP1 foci in mESCs at 24 h post infection of lentiviral sgRNA

Then we identified the frequency of Y chromosome elimination in mESCs with Y chromosome targeting sgRNA infection through single cell expansion and genotyping. The Y-specific gene *Sry* is located on the short arm of mouse Y chromosome (MSYp) (Fig. 1A). Through genotyping, we observed that surviving cells lack of *Sry* were existed in all Y chromosome targeting groups (Fig. S1C). Further confirmation of complete Y chromosome elimination was performed by genotyping of other six Y chromosome genes, located on both short and long arm (Figs. 1A and S1D). Statistically, Y chromosome elimination occurred in all groups of surviving cells infected with different Y chromosome targeting sgRNAs, with a variable efficiency ranging from 40% to 100% according to our result (Fig. S1E). Overall, we demonstrate that CRISPR/Cas9 mediated multiple DNA double strand breaks (DSBs) induce cell death in mESCs (Fig. 1A).

To illustrate the mechanism, we established male or female mESC lines carrying Cas9-T2A-mRFP transgene via PiggyBac transponson mediated insertion and single clone expansion (Fig. S2A). Cas9 expression could be indicated by mRFP fluorescence (Szymczak et al., 2004). In addition, based on the mutation efficiency of different sgRNAs, Ssty1-1, AutoXY-1, and Rosa26-1 were selected as Y chromosome, autosome or X chromosome, and *Rosa26* targeting sgRNA for further study, respectively. Previously, it was reported that CRISPR/Cas9 mediated DNA DSBs induce P53 dependent toxicity in human pluripotent stem cells or hematopoietic stem and progenitor cells (Ihry et al., 2018; Schiroli et al., 2019). Here, we hypothesized that CRISPR/Cas9 mediated multiple DNA DSBs in mESCs activated DNA damage response and triggered P53 dependent cell death. To prove that, firstly the accumulation of 53BP1, a mediator in DNA damage signaling (Polo and Jackson, 2011), were detected in male or female Cas9 expressing mESC lines infected with Ssty1-1, AutoXY-1, or Rosa26-1 respectively at 24 h post infection. According to the result, increased 53BP1 signals were observed in Ssty1-1 infected male compared with female mESCs, reflecting higher DNA damage response burden (Figs. 1D and S3A). Meanwhile, Y chromosome targeting foci-bearing male mESCs showed more aggregated signals in comparison with autosome targeting ones, which presented more scattered spots (Fig. S3A and S3B). For most *Rosa26* targeting foci-bearing male mESCs, one or two 53BP1 foci were observed as expected (Figs. 1D and S3A).

Next, based on the male mESC line carrying Cas9-T2A-mRFP transgene, we generated two P53 knockout Cas9 expressing male mESC lines through CRISPR/Cas9 mediated transient transfection and single clone expansion (Fig. S2B). Western blot confirmed the complete P53 deficiency (Fig. S2C). Then, established P53 knockout Cas9 expressing male mESC lines were infected with Ssty1-1 and stained with 53BP1 antibody. Of note, according to 53BP1 signal, P53 deficiency did not impact the formation of DNA damage response foci induced by CRISPR/Cas9, which was consistent with previous research on human hematopoietic stem cells (Schiroli et al., 2019). Moreover, in contrast to single 53BP1 focus in P53 wild-type mESCs, double 53BP1 foci were observed in P53 knockout mESCs, implying mitigating effect of G_1_ arrest (Fig. S3A and S3C). Furthermore, cell proliferation was analyzed in wild-type female or P53 knockout male Cas9 expressing mESC lines infected with Ssty1-1, Ssty2-3, AutoXY-1, or Rosa26-1 at 0, 24, 48, 72 h post infection respectively (Fig. S2D and S3D). Male mESCs infected with Y chromosome targeting sgRNAs presented significantly decreased cell proliferation and survival compared with female mESCs. Moreover, P53 deficiency partially rescued the impaired cell proliferation, indicating involvement of P53 in this process. For Cas9 expressing mESCs infected with AutoXY-1, both male and female mESCs died at 72 h post infection. However, in contrast to wild-type mESCs whose survival decreased with time, P53 deficient mESCs proliferated at the first 24 h and then all died in the following 48 h. In addition, male or female mESCs infected with Rosa26-1, wild-type or knockout P53 proliferated with no significant difference.

Furthermore, we conducted high-throughput RNA-sequencing in wild-type male or female Cas9-expressing mESC lines infected with Ssty1-1, Ssty2-3, AutoXY-1, or Rosa26-1 at 24, 36 h post infection respectively. Venn diagram showed that differentially expressed genes (DEGs) between those mESC lines above (Fig. S4A). The results of high-throughput sequencing are plotted as a heat map (Fig. S4B). Subsequently, KEGG pathway analysis revealed that targeting repetitive sequence in mESCs resulted in significant variations of genes involved in P53 mediated cell death or apoptosis signaling pathway (Fig. S4C and S4D). Overall, those confirmed that multiple DNA DSBs induced by CRISPR/Cas9 triggered DNA damage response and led to P53 dependent cell death in mESCs.

Previously, it was reported that *Hspa2* promoted Cas9 expression were specifically detected in mice spermatogenic cells. Mice carrying *Hspa2* promoted Cas9 and constitutively expressed sgRNA targeting *Sycp3* exhibited spermatogenic cell specific *Sycp3* gene knockout (Bai et al., 2016). Here, we hypothesize that *Hspa2* promoted Cas9 mediated Y chromosome targeting in mice could realize Y chromosome carrying gametes elimination during spermatogenesis (Fig. 2A). To explore that, we firstly generated mice carrying constitutively expressed Ssty1-1 sgRNA targeting repetitive sequences on Y chromosome through cytoplasmic injection of androgenetic haploid ESC into MII oocyte (Fig. 2B) (Zhong et al., 2015). Then, we crossed *Hspa2* promoted Cas9 expressing mice (*Hspa2*::Cas9) with mice carrying constitutively expressed Ssty1-1 (*U6*::*Ssty1-1)* (Fig. 2B). Genotyping analysis indicated that four progenies carried both *Hspa2*::Cas9 and *U6*::*Ssty1-1* transgenes, half of which were male (Fig. 2C). Next, we crossed mice with wild-type female mice and analyzed sex ratio of progenies. Compared with wild-type, the progeny of *Hspa2*::Cas9;*U6*::*Ssty1-1* male mice exhibited more females than males (Fig. 2D; Table S2). Moreover, while the sex ratio was female-biased, the total number of progenies were no significant difference between wild-type and *Hspa2*::Cas9;*U6*::*Ssty1-1* male mice (Fig. 2E; Table S2).

**Figure 2:**
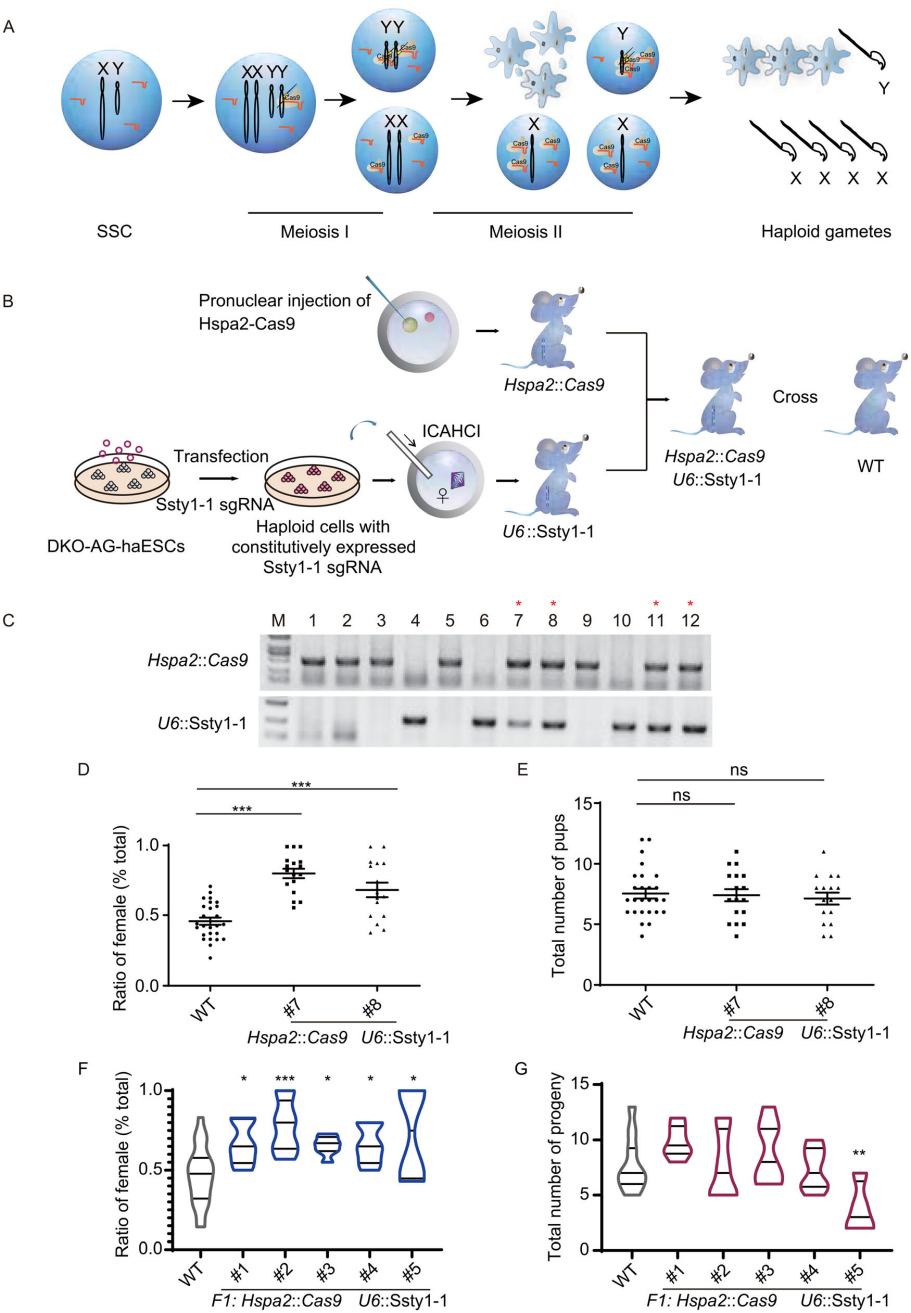
**Female-biased gender control through CRISPR/Cas9 mediated spermatogenic cell specific Y chromosome targeting in mice**. (A) Schematic of CRISPR/Cas9 mediated Y-chromosome-carrying spermatogenic cell death. (B) Scheme illustrating generation of mice carrying *Hspa2* promoted Cas9 and sgRNA targeting Y chromosome. (C) Genotyping of progenies from crossing mice carrying *Hspa2* promoted Cas9 with Ssty1-1 sgRNA. M: DNA Ladder. (D) Ratio of female in progenies from crossing male carrying *Hspa2* promoted Cas9 and Ssty1-1 sgRNA with wild-type female. Unpaired *t* test; ***P < 0.001. (E) Total number of progenies from crossing male carrying *Hspa2* promoted Cas9 and Ssty1-1 sgRNA with wild-type female. Unpaired *t* test; ns, not significant. (F) Ratio of female in progenies from crossing F1 (first filial generation) male carrying *Hspa2* promoted Cas9 and Ssty1-1 sgRNA with wild-type female. Unpaired *t* test; *P < 0.05; ***P < 0.001. (G) Total number of progenies from crossing F1 (first filial generation) male carrying *Hspa2* promoted Cas9 and Ssty1-1 sgRNA with wild-type female. Unpaired *t* test; **P < 0.01

Furthermore, we crossed *Hspa2*::Cas9;*U6*::*Ssty1-1* female mice with wild-type males. And all the *Hspa2*::Cas9;*U6*::*Ssty1-1* male progenies were further crossed with wild-type female mice. We demonstrated that the progenies from all the five *Hspa2*::Cas9;*U6*::Ssty1-1 male mice (first filial generation, F1) presented significantly more females than males compared with wild-type mice (Fig. 2F; Table S3). However, the total number of progenies from four *Hspa2*::Cas9;*U6*::*Ssty1-1* male mice (F1) were no significant difference compared with wild-type mice. One *Hspa2*::Cas9;*U6*::Ssty1-1 male mice (F1) showed significantly decreased number of progenies, probably due to large interstitial MSYq deletions (Fig. 2G; Table S3) (Conway et al., 1994; Inselman et al., 2010; Styrna et al., 2002). Meanwhile, twenty female progenies (F1) were analyzed for the copy number of X chromosome through real-time PCR. Eighteen of twenty female progenies carried two copies of X chromosome, while two carried only one copy of X chromosome, possibly due to CRISPR/Cas9 mediated targeted chromosome elimination (Fig. S5A) (Zuo et al., 2017). Moreover, we collected haploid, diploid and tetraploid spermatogenic cells from *Hspa2*::Cas9;*U6*::Ssty1-1 male mice using flow cytometry (Fig. S5B and S6A) (Gaysinskaya et al., 2014). Immunofluorescence staining confirmed the phase specific isolation (Fig. S6B). Through real-time PCR, we detected the copy number of X and Y chromosome in spermatogenic cells during meiosis. The result illustrated that along with meiosis copy number of Y chromosome in *Hspa2*::Cas9;*U6*::*Ssty1-1* males showed consistent reduction compared with wild-type mice, implying Y chromosome carrying spermatogenic cell death during spermatogenesis (Fig. S5C and S6C).

In summary, we provide a first proof of concept that repetitive sequence targeting, instead of particular genes through CRISPR/Cas9 could mediate female biased gender control in mice. Our research also paves the way for further study on evolutionary intervention as well as artificial breeding.

## Supplementary Information

Below is the link to the electronic supplementary material.

Supplementary file1 (PDF 24326 kb)
