# Correction to: Gonadal mosaicism mediated female-biased gender control in mice

**DOI:** 10.1007/s13238-022-00918-2

**Published:** 2022-05-07

**Authors:** Meizhu Bai, Dan Liang, Yan Cheng, Guolong Liu, Qiudao Wang, Jinsong Li, Yuxuan Wu

**Affiliations:** 1 State Key Laboratory of Cell Biology, Shanghai Key Laboratory of Molecular Andrology, CAS Center for Excellence in Molecular Cell Science, Shanghai Institute of Biochemistry and Cell Biology, Chinese Academy of Sciences, University of Chinese Academy of Sciences, Shanghai, 200031 China; 2 Shanghai Frontiers Science Center of Genome Editing and Cell Therapy, Shanghai Key Laboratory of Regulatory Biology, Institute of Biomedical Sciences and School of Life Sciences, East China Normal University, Shanghai, 200241 China; 3 Department of Obstetrics and Gynecology, The First Affiliated Hospital of Anhui Medical University, Hefei, 230022 China; 4 NHC Key Laboratory of Study on Abnormal Gametes and Reproductive Tract (Anhui Medical University), Hefei, 230032 China; 5 Key Laboratory of Population Health Across Life Cycle (Anhui Medical University), Ministry of Education of the People’s Republic of China, Hefei, 230032 China

## Correction to: Protein Cell 10.1007/s13238-022-00910-w

In the original article, there were some errors in the funding part and in this correction, the correct funding text is as follows:

This work was supported by the National Key R&D Program of China (2019YFA0109900, 2019YFA0802800).

The Original article has been corrected.

