# Supplementary material for: Gonadal mosaicism mediated female-biased gender control in mice
Source: Protein Cell. 2022 Mar 25;13(11):863–8. doi: 10.1007/s13238-022-00910-w (PMC9237195; doi:10.1007/s13238-022-00910-w)
Supplement: Supplementary file 1 — Supplementary file1 (PDF 24326 kb) [file 13238_2022_910_MOESM1_ESM.pdf]

Methods and Materials

Ethics Satement

All animal experiment were performed under the ethical guidelines (Approval number: IBCB0070) approved by the Animal Care and Use Committee of Shanghai Institute of Biochemistry and Cell Biology, Chinese Academy of Sciences.

Derivation of mESC lines and genotyping

2-cell embryos were collected from C57BL6/J females crossed with C57BL6/J males. After in vitro culture, morulas or blastocysts were selected to establish ES cell lines. To begin with, the zona pellucida was removed using acid Tyrode solution and embryo was transferred into 96 well plate coated with feeder cells one by one. After cultured in ESC medium for 4-5 days, outgrowth was selected, trypsinized and transferred into a new well coated with feeder cells. Cells were then proceeded with regular clone expansion and genotyping. To identify gender of each cell line, we designed primers on *Sry* and *Mecp2* for Y chromosome and X chromosome detection, respectively. Through polymerase chain reaction (PCR) and electrophoreses, cell lines with both *Sry* and *Mecp2* were male, while with *Mecp2* only were female.

Lentivirus production and mESC infection

SgRNAs targeting Y chromosome, autosome/X chromosome, or *Rosa26* gene were designed, synthesized, annealed, and ligated into pKLV-U6gRNA(BbsI)-PGKpuro2ABFP (Addgene plasmid # 50946) digested with

BbsI (Fermentas). LentiCas9-Blast (Addgene plasmid # 52962) and the constructed sgRNA plasmids were individually transfected into low-passage 293T cells at 80% confluency in 10cm tissue culture plates with psPAX2 and VSVG using polyethyenimine (PEI). Viral supernatant was collected at 72h post-transfection, filtered with 0.45um filtration unit (Millipore), and concentrated through ultracentrifugation. The concentrated supernatant was subsequently aliquoted and stored at -80℃ until use. At the same time, titer of each lentivirus was measured through gradient infection and antibiotic selection.

To establish mESC lines carrying constitutively expressed Cas9, the derived male or female mESCs were infected with lentivirus packaged from LentiCas9-Blast for 2 days and then selected with blasticidin. For lentiviral sgRNA infection, appropriated amount of lentivirus were added to each Cas9 expressing cell line for 2 days according to titer measurement to insure that almost all cells were infected.

CRISPR/Cas9 mediated gene knockout in mESCs

To generate P53 knockout mESC lines, sgRNAs targeting *Trp53* were designed, synthesized, annealed, and ligated to px330-mcherry (Addgene plasmid # 98750) digested with BbsI (Fermentas). Established mESC lines were then transfected with the constructed px330-mcherry plasmid using Lipofectamine 2000 (Thermo Fisher Scientific) according to the manufacturer’s instruction manual. Around 48h after transfection, mcherry positive cells were enriched through flow cytometry (FACS AriaII, BD Biosciences) and then plated at low density. About 4-5 days later, single

colonies were picked up and expansion for further analysis. For genotyping, cells were lysed, PCR amplified of target site and then DNA sequencing.

Cell viability

Cell viability was calculated as the ratio of live cells from each sgRNA expressing group to respective Cas9 only group. Typan blue staining were used to distinguish live/dead cells in each group. Cell counting were performed using Countess Automated Cell Counter (Invitrogen, C10227) according to user manual.

Immunofluorescence staining

Mouse ESCs were trypsinized, washed with DPBS (Thermo Fisher Scientific) and seeded on slides pre-treated with Poly-L-lysine (Sigma-Aldrich). For spermatogenic cells, the enriched cells were seeded on the coated slides directly. Cells were then fixed with 4% paraformaldehyde for 20 min and permeabilized with 0.1% TritonX-100. After blocking with 1% BSA (Sigma-Aldrich), cells were probed with corresponding primary antibodies. After primary antibodies incubation at 4℃ overnight (53BP1 Antibody, Novus Biologicals; Anti-SCP3 antibody, ABcam; Anti-alpha Tubulin antibody, ABcam) (if not specified, the antibody dilution ratio is 1:1000), cells were washed three times with DPBS and incubate with Cy3 (ABcam), Alexa Fluor 488- (Thermo Fisher Scientific) labeled secondary antibodies (if not specified, the antibody dilution ratio is 1:10000) for two hours at room temperature. Nuclear DNA was stained with DAPI for 10min. Slides were mounted and fluorescent images were acquired using Leica SP8 Confocal microscopes.

Protein extraction and immunoblotting

Mouse ESCs were collected and lysed with lysis buffer containing 50 mM Tris-HCl (pH 7.4), 1% Triton X-100, 0.1% NP-40, 150 mM NaCl, 5 mM EDTA, 1× proteinase Inhibitor cocktail (Roche, 4693159001). Total proteins were extracted, quantified and fractionated through SDS-PAGE, followed by transferring to polyvinylidene difluoride membrane using a transfer apparatus according to the manufacturer's protocols. After blocking with blocking buffer (Thermo Fisher Scientific) for 1 hour, the membrane was incubated with atibody against P53 (1:1000); beta-Actin (1:5000) (Cell Signalling technology) at 4℃ overnight. Membranes were washed three times for 10min and incubated with a 1:3000 dilution of horseradish peroxidase-conjugated anti-rabbit or anti-mouse antibodies for 2h. Blots were washed three times for 10min and bands were visualized after adding peroxidase substrate.

Generation of transgenic mouse lines

Mice carrying *Hspa2* promoted Cas9 were introduced in our previous study (Bai et al., 2016). Mice with *U6::Ssty1-1* transgene were generated via injection of transgenic androgenetic haploid embryonic stem cells into MⅡ oocytes according to the publications (Yang et al., 2012; Zhong et al., 2015). Briefly, androgenetic haploid embryonic stem cells were firstly transfected with *U6::Ssty1-1* cassette using PiggyBac transponson mediated genetic insertion. Seven days after transfection, mRFP positive haploid cells were selected through flow cytometry and further expansion for oocyte injection. For intracytoplasmic androgenetic haploid embryonic

stem cell injection, transgenic cells were arrested at M phase and injected into MⅡ oocytes using Piezo-drill micromanipulator. Reconstructed embryos were then activated and transferred into oviduct of pseudopregnant ICR females at 0.5 days post coitum (dpc). At 19.5 dpc, transgenic mice were delivered.

Real-time PCR

Templates for real-time PCR were gDNA extracted from tail of female progenies generated by crossing *Hspa2::Cas9; U6::Ssty1-1* male with wild-type female or haploid, diploid, tetraploid spermatogenic cells isolated from *Hspa2::Cas9; U6::Ssty1-1* male testis. qPCR was performed on Applied Biosystems QuantStudio 5 Real-Time PCR system (Applied Biosystems) using FastStart Universal SYBR Green Master (Rox) (Roche). Primer sequences for real-time PCR were listed in **Table S4**.

Spermatogenic cell sorting

Spermatogenic cell sorting in mice were described in previous publications (Bastos et al., 2005; Gaysinskaya et al., 2014). Briefly, mouse testes were harvested and tunica albuginea was removed to expose the seminiferous tubules. Seminiferous tubules were cut into pieces using surgical scissors and incubated in collagenase IV at 37℃ for 15min, followed by 0.5% trypsin digestion at 37℃ for 5min. The digested cell suspension was then filtered with 70um filter (Millipore) and stained with Hoechst33342 (Thermo Fisher Scientific) and Propidium Iodide (Thermo Fisher Scientific) for 30min at 37℃. Cell sorting based on DNA content, Hoechst Blue, and Hoechst Red were performed to isolated haploid, diploid and tetraploid spermatogenic

cells from mouse testis.

Supplemental References

Bai, M., Liang, D., Wang, Y., Li, Q., Wu, Y., and Li, J. (2016). Spermatogenic Cell-Specific Gene Mutation in Mice via CRISPR-Cas9. J Genet Genomics 43, 289-296.

Bastos, H., Lassalle, B., Chicheportiche, A., Riou, L., Testart, J., Allemand, I., and Fouchet, P. (2005). Flow cytometric characterization of viable meiotic and postmeiotic cells by Hoechst 33342 in mouse spermatogenesis. Cytometry A 65, 40-49.

Gaysinskaya, V., Soh, I.Y., van der Heijden, G.W., and Bortvin, A. (2014). Optimized flow cytometry isolation of murine spermatocytes. Cytometry A 85, 556-565.

Yang, H., Shi, L., Wang, B.A., Liang, D., Zhong, C., Liu, W., Nie, Y., Liu, J., Zhao, J., Gao, X., *et al.* (2012). Generation of genetically modified mice by oocyte injection of androgenetic haploid embryonic stem cells. Cell 149, 605-617.

Zhong, C., Yin, Q., Xie, Z., Bai, M., Dong, R., Tang, W., Xing, Y.H., Zhang, H., Yang, S., Chen, L.L., *et al.* (2015). CRISPR-Cas9-Mediated Genetic Screening in Mice with Haploid Embryonic Stem Cells Carrying a Guide RNA Library. Cell Stem Cell 17, 221-232.

Supplemental Figure Legends

Figure S1. CRISPR/Cas9 mediated Y chromosome targeting in mESCs. (A) Derivation of mESCs from the inner cell mass (ICM) of mouse blastocysts. Left: outgrowth from mouse blastocysts; Right: colony morphology of derived mESCs. Scale bars, 50um. **(B)** Genotyping of established mESC lines via PCR analysis of *Sry* on Y chromosome and *Mecp2* on X chromosome. M1-M3: established male mES cell lines; F1-F3: established female mES cell lines. F: Female genomic DNA; M: Male genomic DNA. **(C)** Genotyping of surviving cells after 72h post infection of lentiviral sgRNA targeting Y chromosome via PCR analysis of *Sry* on Y chromosome and *Mecp2* on X chromosome. **(D)** Genotyping of surviving cells after 72h post infection of lentiviral sgRNA targeting Y chromosome via PCR analysis of *Ddx3y*, *Uba1y*, *Kdm5d*, *Tspy* on MSYp and *Ssty1*, *Ssty2* on MSYq. **(E)** Statistics of Y chromosome elimination in surviving cells after 72h post infection of lentiviral sgRNA targeting Y chromosome. n=24.

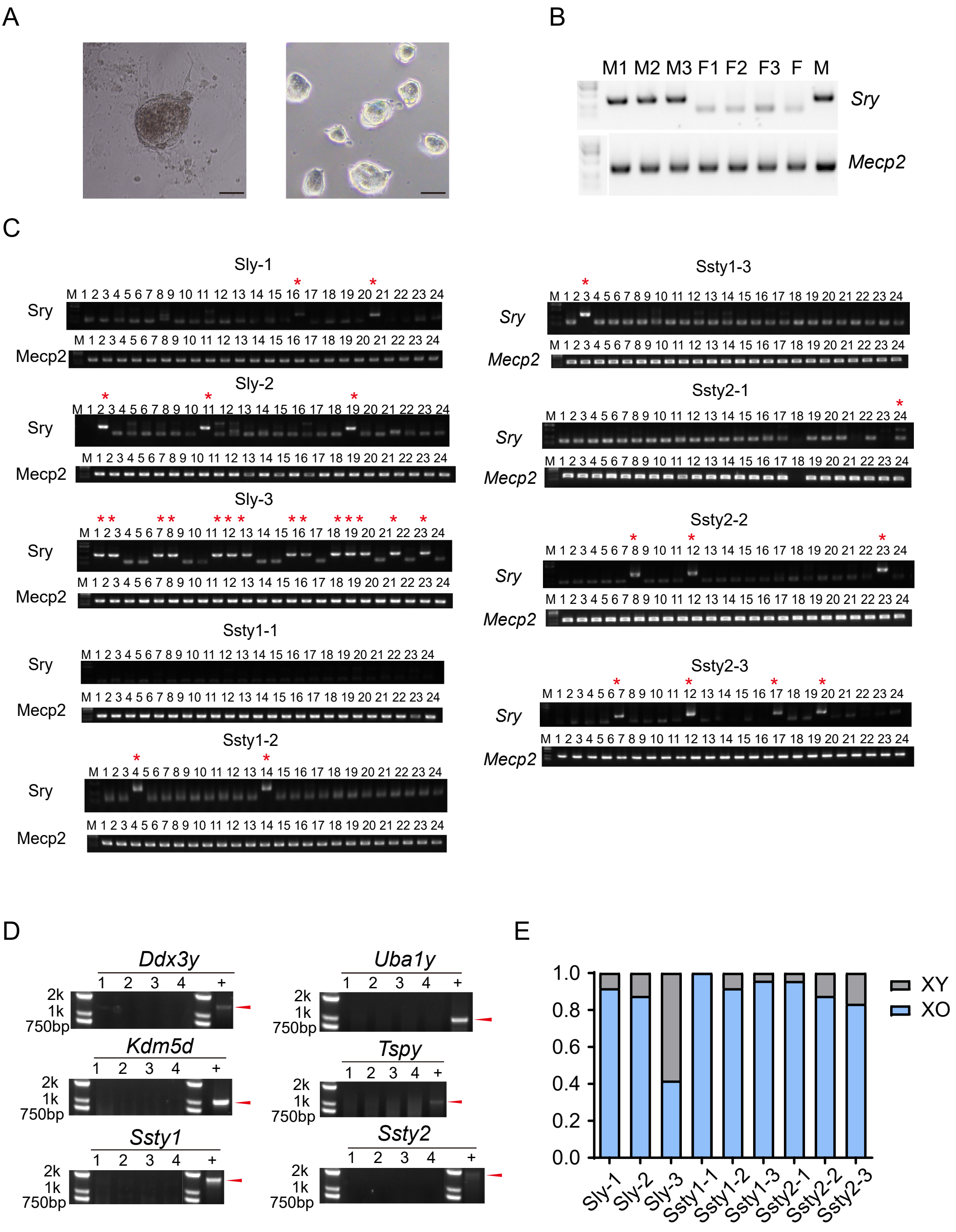

A
B
M1 M2 M3 F1 F2 F3 F M
Sry
Mecp2
C
Sly-1
Sly-2
Sly-3
Ssty1-1
Ssty1-2
Ssty1-3
Ssty2-1
Ssty2-2
Ssty2-3
D
Ddx3y
Uba1y
Kdm5d
Tspy
Ssty1
Ssty2
2k
1k
750bp
E
XY
XO

Figure S2. Cell death induced by CRISPR/Cas9 mediated multiple DNA double strand breaks is P53 dependent. (A) Cas9 expression cassette on PiggyBac transponson. **(B)** Schematic of *Trp53* knockout in mESCs using CRISPR/Cas9 mediated genome editing. **(C)** Western blot confirmation of P53 knockout in two mESC lines. M1: established male mES cell line#1. **(D)** Fluorescence image of surviving cells at 72h post infection of lentiviral sgRNA targeting Y chromosome, autosome, or *Rosa26* gene. M1: established male mES cell line#1; F1: established female mES cell line#1.

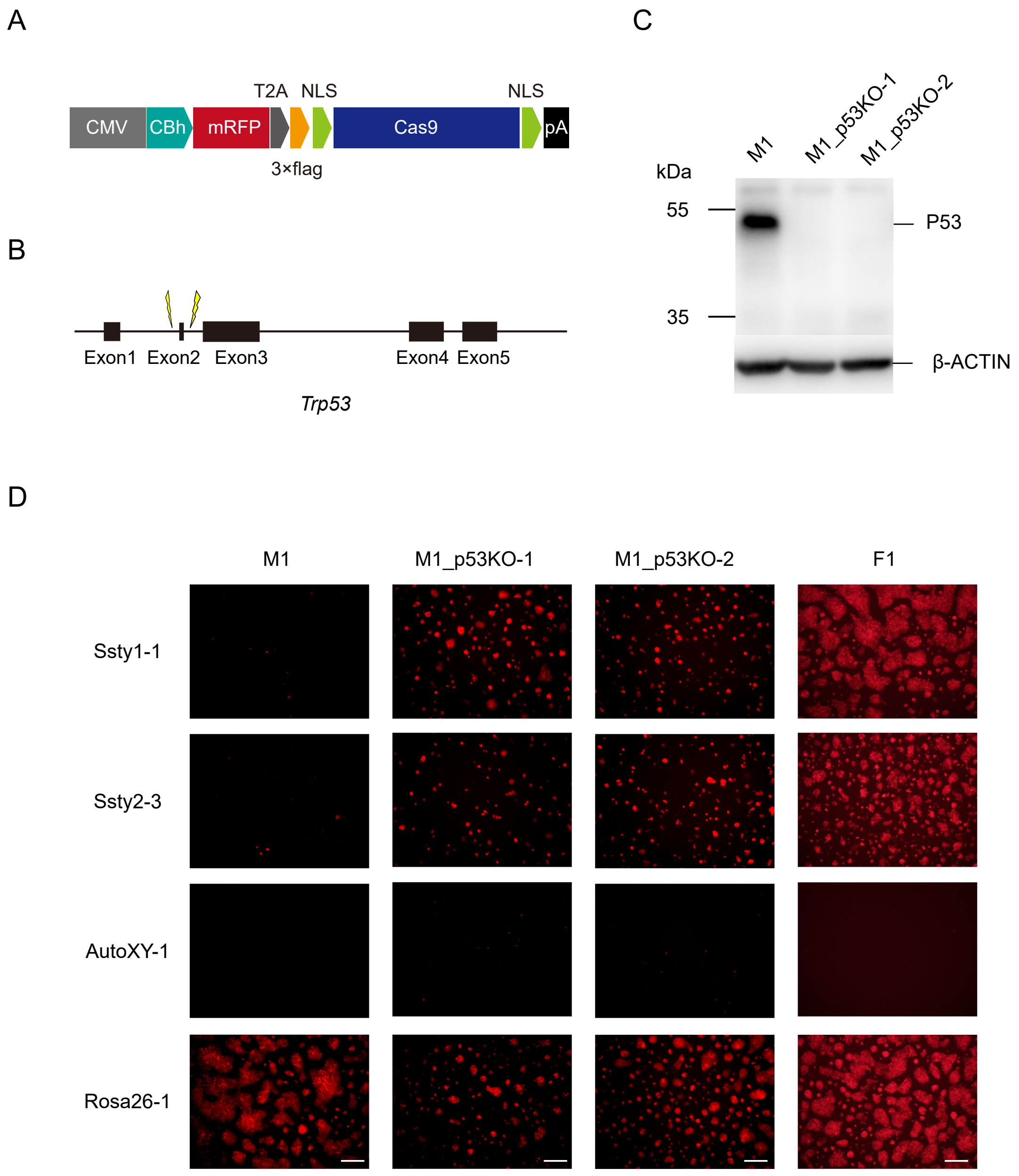
A
CMV
CBh
mRFP
T2A
NLS
Cas9
NLS
pA
3×flag
B
Exon1
Exon2
Exon3
Exon4
Exon5
Trp53
C
M1
M1_p53KO-1
M1_p53KO-2
kDa
55
35
P53
β-ACTIN
D
M1
M1_p53KO-1
M1_p53KO-2
F1
Ssty1-1
Ssty2-3
AutoXY-1
Rosa26-1

Figure S3. Multiple DNA double strand breaks in mESCs induced DNA damage response and P53 dependent cell death. (A) Immunofluorescence staining of 53BP1 in mESCs at 24h post infection of lentiviral sgRNA. Scale bars, 30um. M1: established male mES cell line#1; F1: established female mES cell line#1. **(B-C)** Quantification of $53BP1^{+}$ cells with 1 focus (left), 2 foci (middle) or more than 5 foci (right) in mESCs at 24h post infection of lentiviral sgRNA. Unpaired t test, *P<0.05; ***P<0.001; ****P<0.0001. **(D)** Cell proliferation after infection of lentiviral sgRNA targeting Y chromosome, autosome, or *Rosa26* gene. Two-way ANOVA test, *P<0.05; **P<0.01; ***P<0.001; ns, not significant. M1: established male mES cell line#1; F1: established female mES cell line#1.

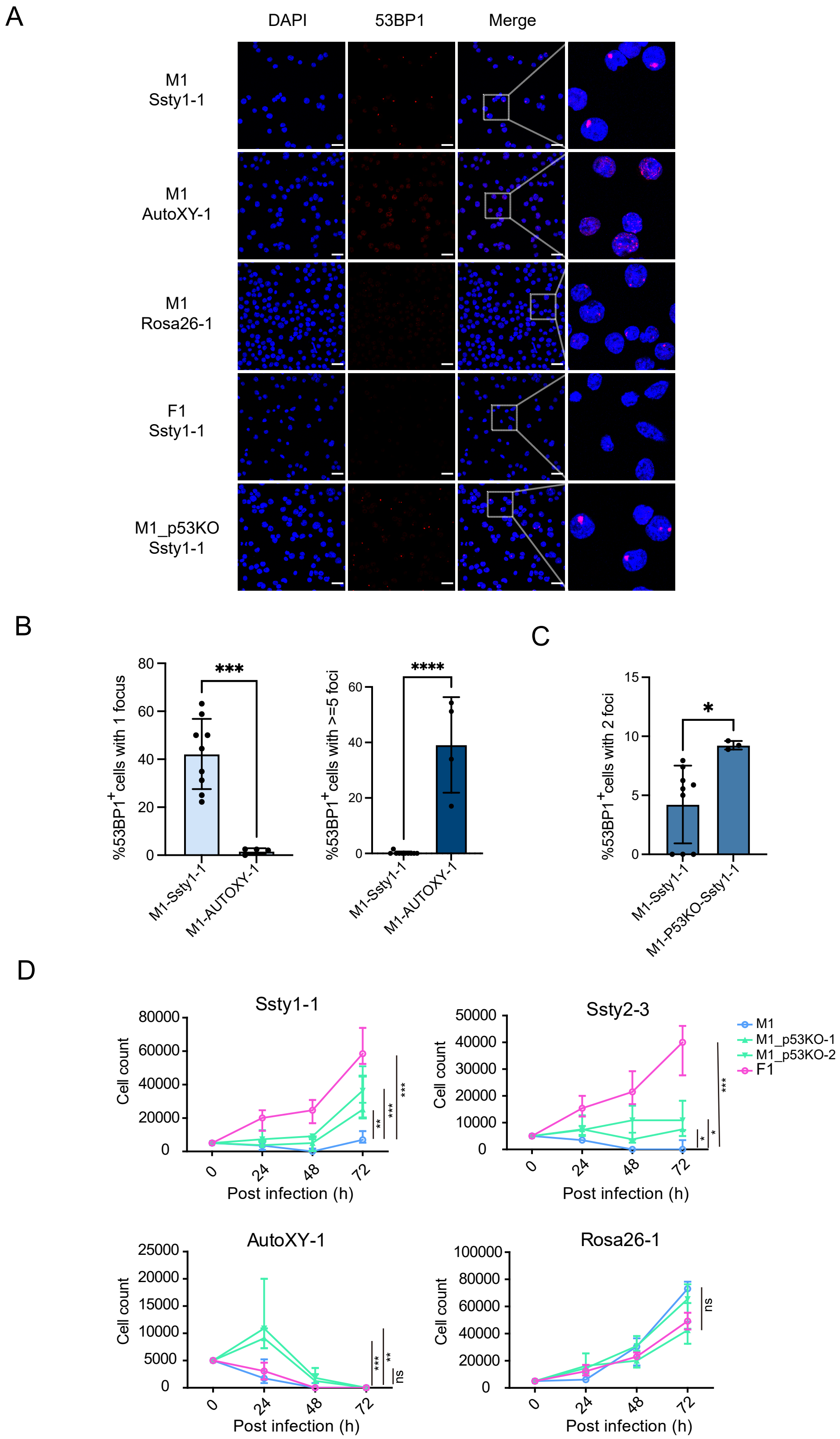

A
DAPI
53BP1
Merge
M1
Ssty1-1
M1
AutoXY-1
M1
Rosa26-1
F1
Ssty1-1
M1_p53KO
Ssty1-1
B
%53BP1+cells with 1 focus
***
M1-Ssty1-1
M1-AUTOXY-1
%53BP1+cells with >=5 foci
****
M1-Ssty1-1
M1-AUTOXY-1
C
%53BP1+cells with 2 foci
*
M1-Ssty1-1
M1-P53KO-Ssty1-1
D
Ssty1-1
Ssty2-3
AutoXY-1
Rosa26-1
Cell count
Post infection (h)
M1
M1_p53KO-1
M1_p53KO-2
F1
ns

Figure S4. Multiple DNA double strand breaks in mESCs induce p53 mediated cell death signaling pathway. **(A)** Venn diagram showing that DEGs between mESCs infected by lentiviral sgRNA targeting Y chromosome, autosome or X chromosome and sgRNAs targeting single locus on *Rosa26* gene. **(B)** Heatmap of RNA-sequencing results in mESCs infected by lentiviral sgRNA targeting Y chromosome, autosome, or *Rosa26* gene. **(C)** KEGG analysis of genes regulated by multiple DNA double strand breaks in mESCs at 24h post infection. **(D)** KEGG analysis of genes regulated by multiple DNA double strand breaks in mESCs at 36h post infection.

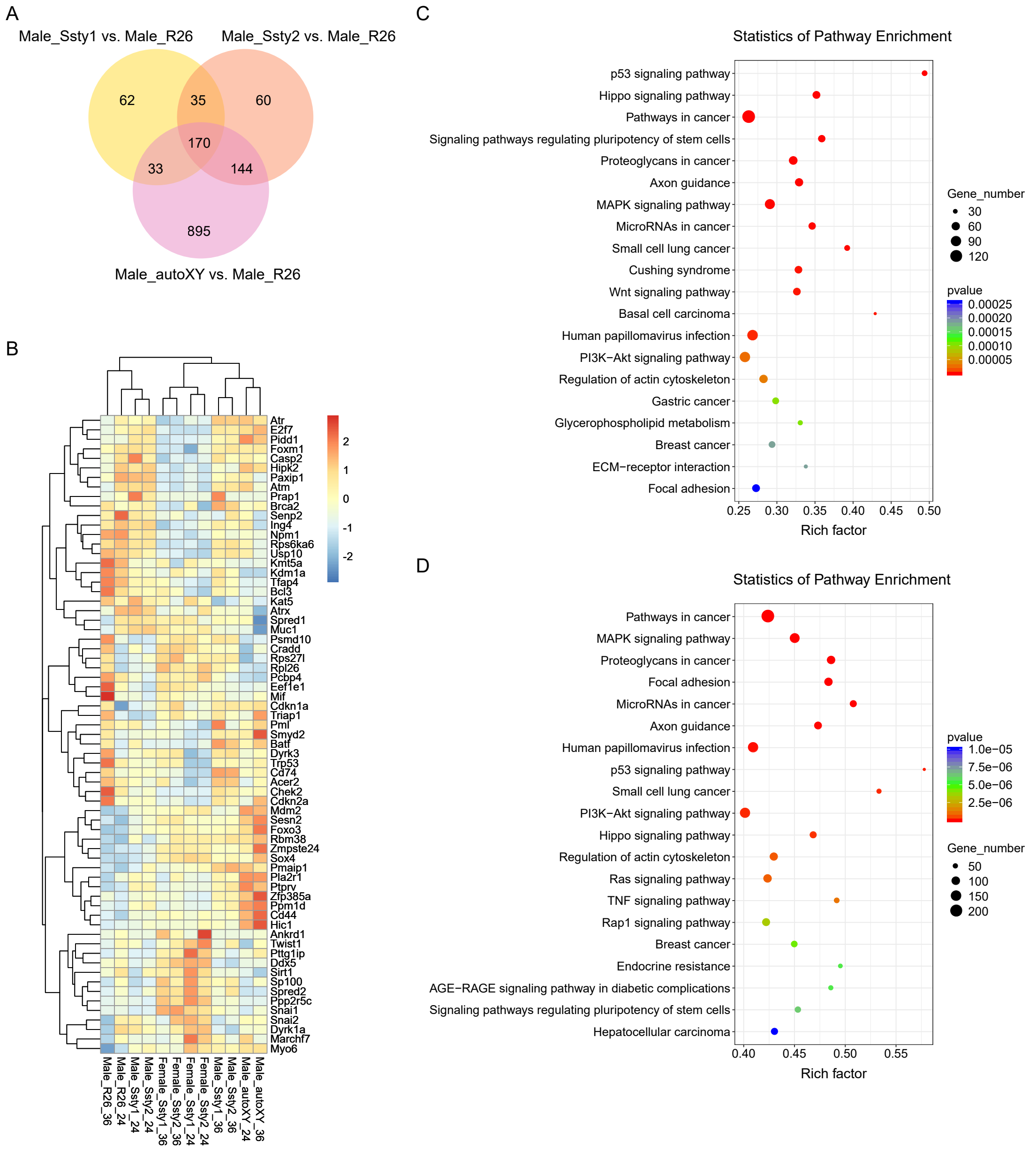
A
Male_Ssty1 vs. Male_R26
Male_Ssty2 vs. Male_R26
Male_autoXY vs. Male_R26
62
35
60
170
33
144
895
B
Atr
E2f7
Pidd1
Foxm1
Casp2
Hipk2
Paxip1
Atm
Prap1
Brca2
Senp2
Ing4
Npm1
Rps6ka6
Usp10
Kmt5a
Kdm1a
Tfap4
Bcl3
Kat5
Atrx
Spred1
Muc1
Psmd10
Cradd
Rps27l
Rpl26
Pcbp4
Eef1e1
Mif
Cdkn1a
Triap1
Pml
Smyd2
Batf
Dyrk3
Trp53
Cd74
Acer2
Chek2
Cdkn2a
Mdm2
Sesn2
Foxo3
Rbm38
Zmpste24
Sox4
Pmaip1
Pla2r1
Ptprv
Zfp385a
Ppm1d
Cd44
Hic1
Ankrd1
Twist1
Pttg1ip
Ddx5
Sirt1
Sp100
Spred2
Ppp2r5c
Snai1
Snai2
Dyrk1a
Marchf7
Myo6
2
1
0
-1
-2
Male_R26_36
Male_R26_24
Male_Ssty1_24
Male_Ssty2_24
Female_Ssty1_36
Female_Ssty2_36
Female_Ssty1_24
Female_Ssty2_24
Male_Ssty1_36
Male_Ssty2_36
Male_autoXY_24
Male_autoXY_36
C
Statistics of Pathway Enrichment
p53 signaling pathway
Hippo signaling pathway
Pathways in cancer
Signaling pathways regulating pluripotency of stem cells
Proteoglycans in cancer
Axon guidance
MAPK signaling pathway
MicroRNAs in cancer
Small cell lung cancer
Cushing syndrome
Wnt signaling pathway
Basal cell carcinoma
Human papillomavirus infection
PI3K−Akt signaling pathway
Regulation of actin cytoskeleton
Gastric cancer
Glycerophospholipid metabolism
Breast cancer
ECM−receptor interaction
Focal adhesion
0.25
0.30
0.35
0.40
0.45
0.50
Rich factor
Gene_number
30
60
90
120
pvalue
0.00025
0.00020
0.00015
0.00010
0.00005
D
Statistics of Pathway Enrichment
Pathways in cancer
MAPK signaling pathway
Proteoglycans in cancer
Focal adhesion
MicroRNAs in cancer
Axon guidance
Human papillomavirus infection
p53 signaling pathway
Small cell lung cancer
PI3K−Akt signaling pathway
Hippo signaling pathway
Regulation of actin cytoskeleton
Ras signaling pathway
TNF signaling pathway
Rap1 signaling pathway
Breast cancer
Endocrine resistance
AGE−RAGE signaling pathway in diabetic complications
Signaling pathways regulating pluripotency of stem cells
Hepatocellular carcinoma
0.40
0.45
0.50
0.55
Rich factor
pvalue
1.0e−05
7.5e−06
5.0e−06
2.5e−06
Gene_number
50
100
150
200

Figure S5. Y chromosome carrying spermatogenic cell elimination during spermatogenesis in mice. (A) Quantitative analysis of X chromosome in F1 (first filial generation) female mice from crossing male carrying *Hspa2* promoted Cas9 and Ssty1-1 sgRNA with wild-type female. **(B)** Spermatogenic cell sorting for haploid, diploid, and tetraploid cells from male testis. **(C)** Quantitative comparison of Y chromosome relative to X chromosome in haploid, diploid, and tetraploid spermatogenic cells isolated from male testis through real-time PCR.

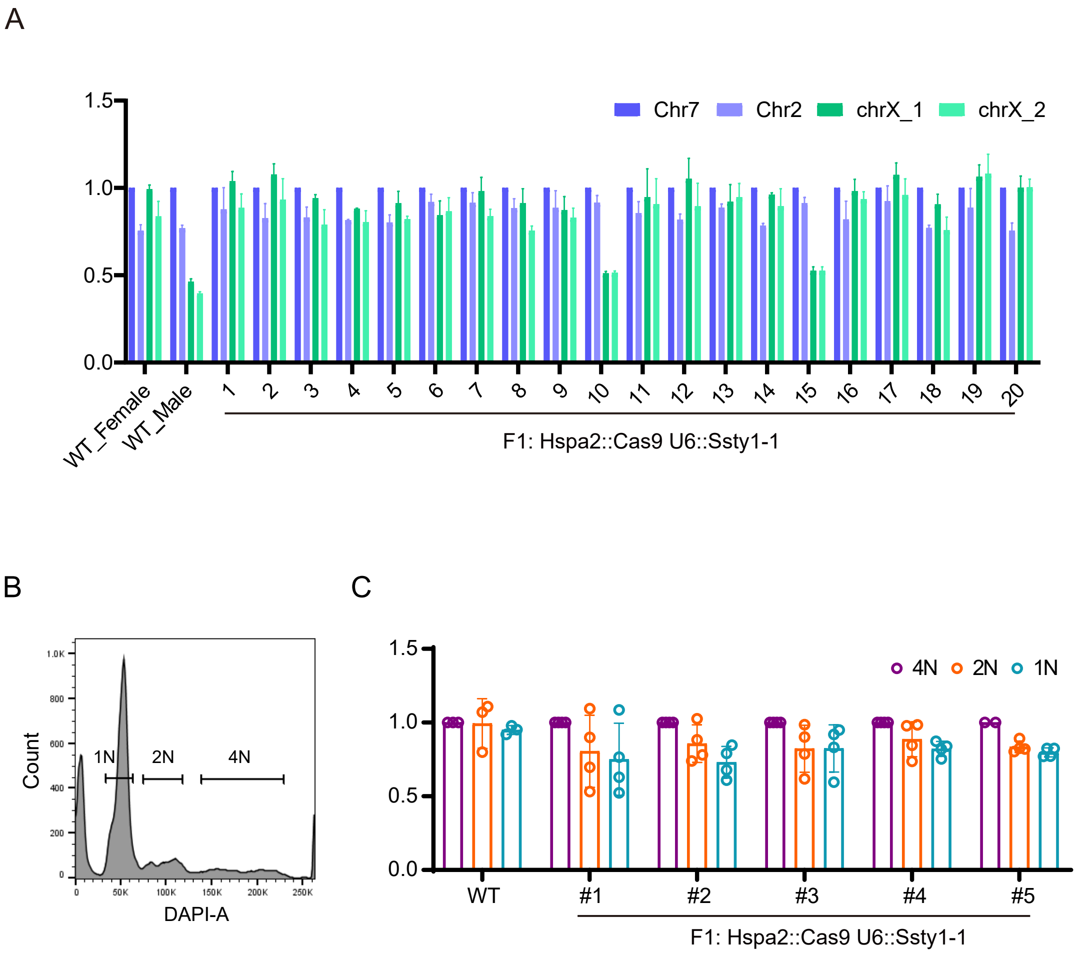
A
1.5
1.0
0.5
0.0
Chr7
Chr2
chrX_1
chrX_2
WT_Female
WT_Male
1
2
3
4
5
6
7
8
9
10
11
12
13
14
15
16
17
18
19
20
F1: Hspa2::Cas9 U6::Ssty1-1
B
Count
1N
2N
4N
DAPI-A
C
1.5
1.0
0.5
0.0
4N
2N
1N
WT
#1
#2
#3
#4
#5
F1: Hspa2::Cas9 U6::Ssty1-1

Figure S6. CRISPR/Cas9 mediated Y chromosome targeting in spermatogenic cells during spermatogenesis in mice. (A) Isolation of haploid, diploid, and tetraploid spermatogenic cells from male testis through flow cytometry. **(B)** Immunofluorescence staining of sorted haploid, diploid, and tetraploid spermatogenic cells using anti-SYCP3, anti-α-TUBULIN, and PNA respectively. **(C)** Quantitative comparison of Y chromosome relative to X chromosome in haploid, diploid, and tetraploid spermatogenic cells isolated from male testis through real-time PCR.

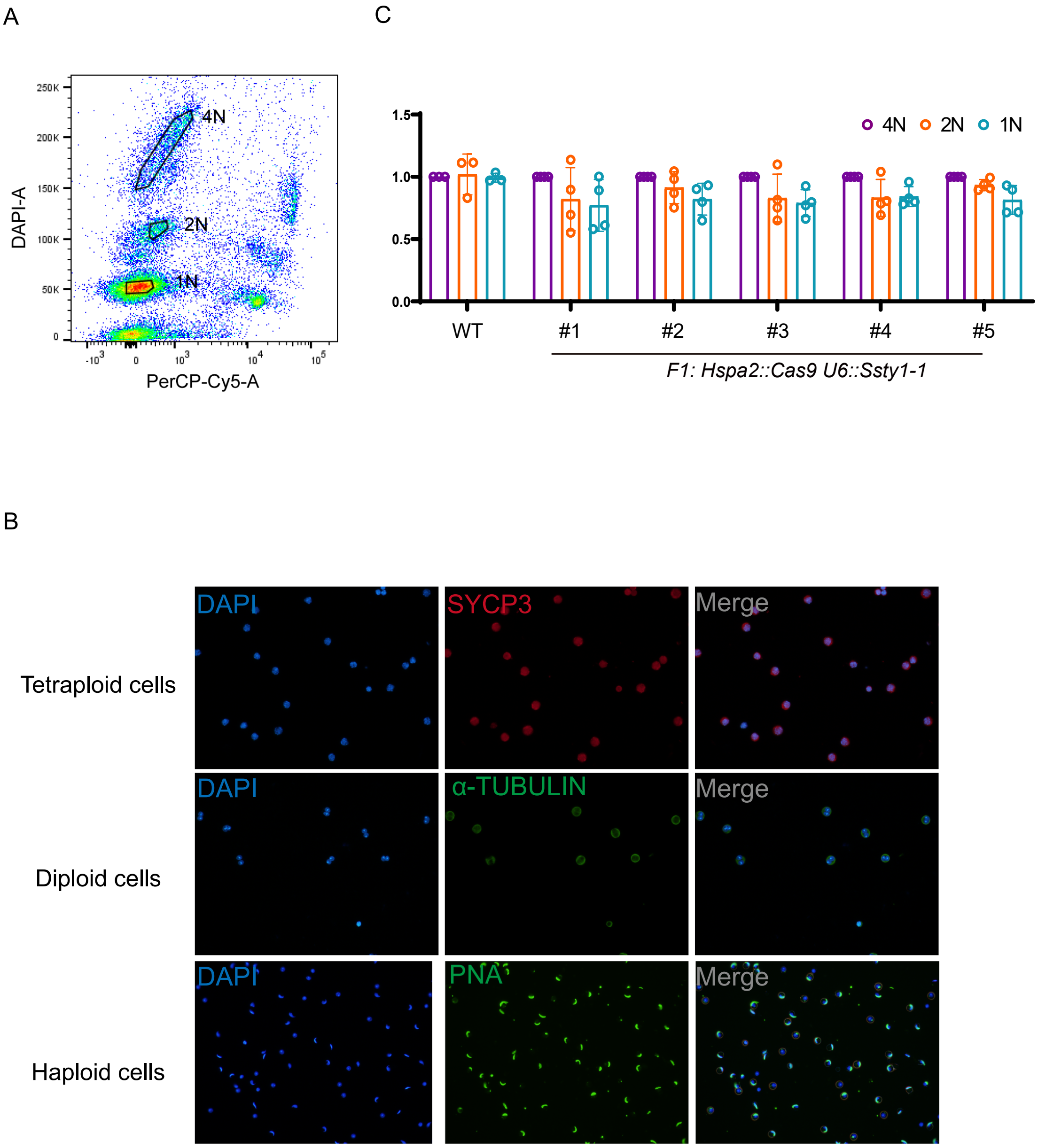

A
250K
200K
150K
100K
50K
0
DAPI-A
4N
2N
1N
-10^3
0
10^3
10^4
10^5
PerCP-Cy5-A
C
1.5
1.0
0.5
0.0
4N
2N
1N
WT
#1
#2
#3
#4
#5
F1: Hspa2::Cas9 U6::Ssty1-1
B
Tetraploid cells
Diploid cells
Haploid cells
DAPI
SYCP3
Merge
DAPI
α-TUBULIN
Merge
DAPI
PNA
Merge

Supplemental Table

Tables S1. Cell viablity ratios of each sgRNA expressing group after 72h transducing into male and female mESCs.

Sex	*Treat with sgRNA*	*Cell viability*										
		1	*2*	*3*	*4*	*5*	*6*	*7*	*8*	*9*	*10*	*11*
mESC_Male	*Sly-1*	0.84	0.82	0.2	0.2	0.28	0.13					
	Sly-2	0.18	0.38	0.1	0.3	0.05	0.34					
	Sly-3	0.05	0.42	0.1	0.2	0.01	0.27					
	Ssty1-1	0.23	0.01	0.1	0	0.14						
	Ssty1-2	0.76	0.83	0.4								
	Ssty1-3	0.32	0.43	0	0.3	0.05	0.23					
	Ssty2-1	0.36	0.25	0.2								
	Ssty2-2	0.3	0.59	0.4	0.1	0.07	0.04	0.08	0.1	0.04	0.05	0.13
	Ssty2-3	0.23	0.57	0.4	0	0.09	0.14	0.08	0	0.09	0.08	0.03
	AutoXY-1	0	0	0								
	AutoX-3	0	0.003	0								
	AutoXY-4	0.003	0	0								
	Rosa26-1	0.81	0.85	0.9								
	Rosa26-2	0.82	0.81	0.9								
mESC_Female	*Sly-1*	0.86	0.79	0.8	0.9	0.83	0.89					
	Sly-2	0.75	0.84	0.8	0.8	0.91	0.81					
	Sly-3	0.77	0.82	0.8	0.8	0.75	0.89					
	Ssty1-1	0.78	0.8	0.9	0.9	0.89	0.75					
	Ssty1-2	0.8	0.81	0.9	0.9	0.76	0.9					
	Ssty1-3	0.78	0.86	0.8	0.8	0.82	0.89					
	Ssty2-1	0.76	0.86	0.9	0.9	0.82	0.87					
	Ssty2-2	0.83	0.79	0.8	0.9	0.84	0.81					
	Ssty2-3	0.85	0.84	0.8	0.9	0.8	0.86					
	AutoXY-1	0	0	0								
	AutoX-3	0.005	0	0								
	AutoXY-4	0.003	0	0								
	Rosa26-1	0.88	0.9	0.8	0.8	0.87	0.82	0.87	0.8	0.79		
	Rosa26-2	0.87	0.78	0.8	0.8	0.87	0.81	0.8	0.8	0.79		

Table S2. Progenies from crossing male carrying *Hspa2* promoted Cas9 and *Ssty1-1* sgRNA with wild-type female.

	WT		Hspa2::Cas9;U6::Ssty1-1			
			#7		#8	
Batch	# of Female	# of Male	# of Female	# of Male	# of Female	# of Male
1	5	4	7	1	7	1
2	3	4	7	0	2	2
3	2	4	4	1	7	0
4	4	3	6	0	4	5
5	3	5	6	1	2	3
6	3	2	6	3	3	5
7	2	4	5	4	5	3
8	5	7	6	4	8	1
9	1	4	5	1	6	1
10	4	7	9	1	3	3
11	4	5	6	1	9	0
12	5	7	8	1	4	2
13	5	2	3	2	7	4
14	2	4	8	3	3	2
15	3	5	6	2	3	1
16	2	5	4	1	7	1
17	5	3	4	0		
18	2	4				
19	3	4				
20	4	2				
21	4	5				
22	2	2				
23	3	3				
24	6	4				
25	4	3				
26	5	3				
Total	91	105	100	26	80	34
	196		126		114	

Table S3. Progenies from crossing F1 male carrying *Hspa2* promoted Cas9 and *Ssty1-1* sgRNA with wild-type female.

	WT		Hspa2::Cas9;U6::Ssty1-1									
			#1		#2		#3		#4		#5	
Batch	# of Female	# of Male	# of Female	# of Male	# of Female	# of Male	# of Female	# of Male	# of Female	# of Male	# of Male	# of Male
1	7	6	6	3	3	2	4	2	8	2	3	4
2	9	4	5	3	7	1	4	2	5	4	2	2
3	5	5	5	4	5	0	8	5	4	2	2	0
4	2	5	5	5	8	2	5	2	5	3	2	0
5	3	3	9	2	5	0	8	3	4	1		
6	5	1	10	2	8	4	6	5	3	3		
7	5	2			4	1	5	3				
8	3	8			4	3						
9	3	3			8	4						
10	2	5										
11	3	2										
12	4	5										
13	5	4										
14	5	6										
15	2	4										
16	4	3										
17	3	5										
18	2	3										
19	5	2										
20	2	3										
21	1	5										
22	1	6										
Total	81	90	40	19	52	17	40	22	29	15	9	6
	171		59		69		62		44		15	

Table S4. Primer sequences for real-time PCR.

**Primer**	**Sequence**
Lenti-Sly sgRNA1-oligoF	CACCGTGACATAAAAGTTTCAGAGGGT
Lenti-Sly sgRNA1-oligoR	TAAAACCCTCTGAAACTTTTATGTCAC
Lenti-Sly sgRNA2-oligoF	CACCGCGATGACATAAAAGTTTCAGGT
Lenti-Sly sgRNA2-oligoR	TAAAACCTGAAACTTTTATGTCATCGC
Lenti-Sly sgRNA3-oligoF	CACCGCCATGGAGGTATGTTGCACAGT
Lenti-Sly sgRNA3-oligoR	TAAAACTGTGCAACATACCTCCATGGC
Lenti-Ssty1 sgRNA1-oligoF	CACCGCATCCCTCATGAAGAAGAGGGT
Lenti-Ssty1 sgRNA1-oligoR	TAAAACCCTCTTCTTCATGAGGGATGC
Lenti-Ssty1 sgRNA2-oligoF	CACCGTTTTACATTACCTACAAGAGT
Lenti-Ssty1 sgRNA2-oligoR	TAAAACTCTTGTAGGTAATGTAAAAC
Lenti-Ssty1 sgRNA3-oligoF	CACCGTTGTACCGCTCTGCCAACCAGT
Lenti-Ssty1 sgRNA3-oligoR	TAAAACTGGTTGGCAGAGCGGTACAAC
Lenti-Ssty2 sgRNA1-oligoF	CACCGTTGGCTGCAGAATTTCTCAGT
Lenti-Ssty2 sgRNA1-oligoR	TAAAACTGAGAAATTCTGCAGCCAAC
Lenti-Ssty2 sgRNA2-oligoF	CACCGTACTTCACCAAATAAAGAGAGT
Lenti-Ssty2 sgRNA2-oligoR	TAAAACTCTCTTTATTTGGTGAAGTAC
Lenti-Ssty2 sgRNA3-oligoF	CACCGTGAGGGGAAAGATGGCTCTGGT
Lenti-Ssty2 sgRNA3-oligoR	TAAAACCAGAGCCATCTTTCCCCTCAC

Lenti-AutoXY-1 sgRNA-oligoF	CACCGAACGCCAGGGCCAAAAAGGGT
Lenti-AutoXY-1 sgRNA-oligoR	TAAAACCCTTTTTGGCCCTGGCGTTC
Lenti-AutoX-3 sgRNA-oligoF	CACCGTAACTCCAGAGATTACCAGAGT
Lenti-AutoX-3 sgRNA-oligoR	TAAAACTCTGGTAATCTCTGGAGTTAC
Lenti-AutoX-4 sgRNA-oligoF	CACCGAGATGGCGAAAGGTAAACGGGT
Lenti-AutoX-4 sgRNA-oligoR	TAAAACCCGTTTACCTTTCGCCATCTC
Lenti-Rosa26-1 sgRNA-oligoF	CACCGGCAGGCTTAAAGGCTAACCGT
Lenti-Rosa26-1 sgRNA-oligoR	TAAAACGGTTAGCCTTTAAGCCTGCC
Lenti-Rosa26-2 sgRNA-oligoF	CACCGCGCCCATCTTCTAGAAAGACGT
Lenti-Rosa26-2 sgRNA-oligoR	TAAAACGTCTTTCTAGAAGATGGGCGC
Sry check outer 1F	GCACATTTTGGTCAGTGGCT
Sry check outer 1R	GCTCTACTCCAGTCTTGCCT
Sry check inner 1F	GTTCAGCCCTACAGCCACATG
Sry check inner 1R	GCAGGCTGTAAAATGCCACTC
Mecp2 check outer 1F	ATAACTGGGCCAAACTGTGC
Mecp2 check outer 1R	GAGTCGCACATCTGTCTGGA
Mecp2 check inner 1F	TGCAGCTTCAGTTCACCTTG
Mecp2 check inner 1R	CCACTAACCACAGGCTCCAT
U6-sg check 1F	GAGGGCCTATTTCCCATGAT
U6-sg check 1R	GCACCGACTCGGTGCCACTT

Uba1y check 1F	CCTCATCACATCACCTCTTCTG
Uba1y check 1R	CTCACTTGCTCCAATCTTCACA
Ddx3y check 1F	ACTCGTTACACTCGTCCTACTC
Ddx3y check 1R	CTTAGCCAGTCCAATCTCTATCAG
Kdm5d check 1F	CACTGAGAAGAAGATAGCCTAG
Kdm5d check 1R	CTGAGAACCACTGATACATGAG
Tspy check 1F	AGGTGAGTTGGAATTGTGTCTG
Tspy check 1R	TGGAGAAGTGTGAAGTTGAAGG
Ssty1 check 1F	GCCACTATAGCTGGATTATGAG
Ssty1 check 1R	GTCTTCACATCAGAGGTTCTAC
Ssty2 check 1F	ACTCACTGTGTAGACCAGACTAG
Ssty2 check 1R	TCCAATTCCTTGCTCACTATGC
Trp53 sgRNA1 oligoF	CACCGACACTCGGAGGGCTTCACT
Trp53 sgRNA1 oligoR	AAACAGTGAAGCCCTCCGAGTGTC
Trp53 sgRNA2 oligoF	CACCGATATCTTCTGGAGGAAGTC
Trp53 sgRNA2 oligoR	AAACGACTTCCTCCAGAAGATATC
Hspa2-c9 check F	CTAGATATCAGTTGGACCACCGGC
Hspa2-c9 check R	TTCTTGCTGGGCACCTTGTACT
chrY qPCR 1F	GTGTGCATCCCTGTGAACGGA
chrY qPCR 1R	CAATGGCACCTGCACCTACCT

chrY qPCR 2F	ACTCAGCCAAGCCCAGTCCA
chrY qPCR 2R	CTGCACAGACCTGCCCACAT
chr7 qPCR F	CGTGTGTGGGCCTTATCTGT
chr7 qPCR R	GCTGGAGGGCTTTTAGGGAG
chr2 qPCR F	CAAGTCACCAACCAGCACCT
chr2 qPCR R	ACAGTCCCAATCAGCATGGC
chrX qPCR 1F	GCCAAGGGTAGCAAGAGATGA
chrX qPCR 1R	CTAAGCTGTTAGTGCCGTCCA
chrX qPCR 2F	GCTTGGTGCCCTACCATTCT
chrX qPCR 2R	GGCTGCTTCACAGATGGAGA
